# Moderate Consumption of Beer (with and without Ethanol) and Menopausal Symptoms: Results from a Parallel Clinical Trial in Postmenopausal Women

**DOI:** 10.3390/nu13072278

**Published:** 2021-06-30

**Authors:** Marta Trius-Soler, María Marhuenda-Muñoz, Emily P. Laveriano-Santos, Miriam Martínez-Huélamo, Gemma Sasot, Carolina E. Storniolo, Ramon Estruch, Rosa M. Lamuela-Raventós, Anna Tresserra-Rimbau

**Affiliations:** 1 Department of Nutrition, Food Sciences and Gastronomy, XaRTA, School of Pharmacy and Food Sciences, University of Barcelona, 08028 Barcelona, Spain; mtrius@ub.edu (M.T.-S.); mmarhuendam@ub.edu (M.M.-M.); elaversa21@alumnes.ub.edu (E.P.L.-S.); mmartinezh@ub.edu (M.M.-H.); gemmamsf@gmail.com (G.S.); carolina.e.storniolo@gmail.com (C.E.S.); 2 INSA-UB, Nutrition and Food Safety Research Institute, University of Barcelona, 08921 Santa Coloma de Gramanet, Spain; 3 CIBER Fisiopatología de la Obesidad y Nutrición (CIBEROBN), Instituto de Salud Carlos III, 28029 Madrid, Spain; restruch@clinic.cat; 4 Department of Internal Medicine, Hospital Clínic, Institut d’Investigacions Biomèdiques August Pi i Sunyer (IDIBAPS), University of Barcelona, 08036 Barcelona, Spain

**Keywords:** phytoestrogens, prenylflavonoids, polyphenols, health, menopause, alcohol, cardiovascular risk factors

## Abstract

The menopausal transition can be a challenging period for women’s health and a trigger of uncomfortable symptoms. Beer is the main food source of isoxanthohumol, a precursor of 8-prenylnaringenin, the strongest phytoestrogen identified to date. As phytoestrogens are reported to reduce perimenopausal symptoms, we evaluated if a daily moderate consumption of beer with (AB) and without alcohol (NAB) could improve menopausal symptoms and modify cardiovascular risk factors. A total of 37 postmenopausal women were enrolled in a parallel controlled intervention trial and assigned to three study groups: 16 were administered AB (330 mL/day), 7 NAB (660 mL/day), and 14 were in the control group. After a 6-month follow-up of the 34 participants who finished the trial, both interventions (AB and NAB) significantly reduced the severity of the menopause-related symptoms (*p*-value AB vs. Control: 0.009; *p*-value NAB vs. Control: 0.033). Moreover, AB had a beneficial net effect on psychological menopausal discomforts compared to the control group. As the sex hormone profile did not differ significantly between the study groups, the effects of both types of beers (AB and NAB) are attributed to the non-alcoholic fraction of beer. Furthermore, moderate NAB consumption improved the lipid profile and decreased blood pressure in postmenopausal women.

## 1. Introduction

Menopause is characterized by a low output of ovarian estrogens and a high production of pituitary gonadotropin hormones (follicle-stimulating hormone (FSH) and luteinizing hormone (LH)), which trigger uncomfortable symptoms such as hot flashes, night sweats, sleep disturbance, and vaginal dryness [1,2,3]. From a health point of view, estrogens are widely regarded as protectors against atherosclerosis, whereas progesterone and androgens may act as atherogenic factors in postmenopausal women [4]. A smooth transition through this challenging period is considered crucial for healthy and successful aging [2]. Although hormone replacement therapy effectively reduces vasomotor symptoms associated with the decrease of estrogen, its benefits do not outweigh the higher risks of stroke and venous thromboembolism or the increased incidence of breast cancer and coronary heart events associated with the co-administration of estrogens and progestin [5]. A post-hoc analysis of data from the Women’s Health Initiative and some new evidence indicate that hormone replacement therapy may have beneficial health effects for women below the age of 60, which calls for a reevaluation of the use of estrogen alone in younger postmenopausal women [6,7]. The inconclusive nature of these results has generated great interest in alternative therapies, such as phytoestrogens, to relieve menopausal symptoms [8,9].

Phytoestrogens are compounds with estrogen-like properties naturally found in plants [7,10,11]. Among flavonoids, isoflavones are the subclass with the highest phytoestrogen effect [12]. In recent years, prenylated chalcones, flavonoids present in hops (*Humulus lupulus* L.), have attracted considerable attention for their health benefits [13,14,15,16,17,18]. Beer is the main dietary source of isoxanthohumol (IX), which is produced from xanthohumol (XN) during the brewing process [19]. Once ingested, the weakly estrogenic IX can be bioactivated to 8-prenylnaringenin (8-PN), the strongest phytoestrogen identified to date [20,21], by microorganisms inhabiting the gastrointestinal tract [17,22] or converted in the liver in minor amounts [17,23,24]. In a previous intervention study with 36 menopausal women, Erkkola et al. (2010) observed that 100 µg/day of 8-PN from a hop extract relieved the symptoms of menopause and increased the quality of life of menopausal women [13]. Our research group has determined that Spanish beers contain around 500 µg/L of IX and 50 µg/L of 8-PN [25].

Accordingly, we conducted a six-month parallel, controlled clinical intervention trial to evaluate if a moderate daily intake of beer, with or without alcohol, could reduce menopausal discomforts in women going through the menopause transition. The effect of the intervention on the female sex hormone profile and cardiovascular risk factors (CVRF) was also monitored.

## 2. Materials and Methods

### 2.1. Study Population and Recruitment

Postmenopausal women aged 45–70 years were recruited into the study between April 2017 and June 2019 from the Outpatient Clinic of the Internal Medicine Department of the Hospital Clinic of Barcelona through poster boards in different settings and advertisements on the radio. Potential participants were screened according to the defined inclusion and exclusion criteria. The postmenopausal status of each participant was validated by the following criteria: (1) absence of menses for the last 12 months; (2) follicle-stimulating hormone (FSH) blood levels of 23–116 U/L, and (3) 17-β-estradiol (E2) blood levels < 37 pg/mL. Women using estrogen therapy or taking silicon or polyphenol supplements were excluded.

### 2.2. Study Design

The participants were assigned to one of the three study groups according to their preference, given that the intervention involved a medium alcohol consumption. The non-randomized design was chosen in accordance with ethical considerations but also reflecting the reality of participant lifestyle.

After a run-in period of 15 days, in which subjects were asked not to consume any alcoholic beverage, NAB or hop-related products, participants were allotted to a study group for 6 months. One group consumed 14 g of ethanol a day in the form of AB (330 mL/d) (AB group); another received NAB (660 mL/d) containing a similar amount of non-alcoholic compounds to AB (NAB group), and the third group did not receive any intervention and were instructed to refrain from consuming alcohol, NAB or hop-related products (control group). None of the participants were allowed to consume any other alcoholic beverages during the study.

For a parallel design, the sample size calculation indicated that to detect mean differences of 3 points in Menopause Rating Scale (MRS) total score with a standard deviation (SD) of 3 points assuming a maximum loss of 10% of participants, 18 subjects per group were needed to complete the study (α = 0.05; power = 80%).

All participants signed an informed consent. Eligible subjects were asked to come for four visits during the intervention period (baseline, and 1.5, 3, and 6 months). The study protocol was approved by the Bioethics Commission of the University of Barcelona (Institutional Review Board: IRB 00003099) in March 2017, registered at ISRCTN (ISRCTN14959650) and conducted in compliance with the Declaration of Helsinki.

### 2.3. Intervention-Phytoestrogen Dose

To standardize the daily phytoestrogen dose for each group, participants consumed the same brand of beer throughout the study and were encouraged to drink it during meals. They were supplied with beers every month as a measure of intervention compliance. The contents of prenylflavonoids IX, 8-PN and XN are detailed in Table 1. Due to the dietary nature of the intervention, neither the participants nor the researchers were blind to the intervention treatments. However, laboratory personnel and technicians were blinded to the intervention received by the participants. Intervention compliance was assessed by data obtained from structured questionnaires and by the measurement of IX, a validated beer intake biomarker, in 24-h urine samples collected at baseline, and 1.5, 3, and 6 months by solid phase extraction liquid chromatography coupled to mass spectrometry (LC-MS/MS) [25]. 

#### Quantification of Prenylflavonoids in Beer Intervention Samples by LC-MS/MS

Qualitative and quantitative analyses of prenylflavonoids in beer samples were carried out according to the method of Quifer-Rada et al. (2013), with some modifications [26]. Briefly, the beer foam from AB and NAB was removed by agitation and ultrasonication. Then, the alcohol content from alcoholic beer was evaporated under a gentle stream of N_2_ and was refilled with water. Samples were filtered through a 0.45-µm polytetrafluoroethylene filter and 600 ng/mL of taxifolin was added as an internal standard prior to the analysis. The identification and quantification of the selected analytes (IX, XN, 6-prenylnaringenin (6-PN), and 8-PN) was carried out using an Acquity UHPLC system equipped with a Waters binary pump (Waters, Milford, MA, USA). The UHPLC separation was performed with a Luna C18 column, 50 mm × 2.0 mm i.d., 5 μm (Phenomenex, Torrance, CA, USA), directly interfaced to an API 3000™triple quadrupole mass spectrometer (Sciex, Concord, ON, Canada) with a turbo ion spray source working in negative mode. The mobile phases used were 5 mM of ammonium bicarbonate buffer adjusted to pH 7.0 and acetonitrile and methanol (1:1), at a constant flow rate of 600 µL/min and a column temperature of 40 °C. Sample injection volume was 10 µL. Multiple reaction monitoring mode was used to identify and quantify the analytes. Calibration curves from 0 to 1000 ppb were prepared adding standards to pure water containing 20 mg/L of ascorbic acid. The reagents, materials, and MS/MS parameters were the same as reported in Quifer-Rada et al. (2013) [18].

### 2.4. Measurements and Outcome Assessment

#### 2.4.1. Medical History

Individual information was collected at baseline and updated through the trial by face-to-face interviews. Interviews were based on a structured questionnaire that included medical and sociodemographic questions, with special attention given to menopausal symptoms and CVRF. Current and past consumption of alcohol, smoking and sleeping habits, daily life and work stress, time since the onset of menopause, and medication received were also recorded. 

#### 2.4.2. Climacteric Symptoms

The primary outcome was changes in menopausal symptomatology. Menopausal discomforts were quantified (frequency and severity) using the MRS questionnaire [1,27,28]. The MRS consists of three independent factorial dimensions, with four items in the somato-vegetative subscale, four items in the psychological subscale and three additional items in the urogenital subscale. Each of the eleven symptoms were rated from 0 (no complaints) to 4 (very severe symptoms) as perceived by the participant, with a total MRS score ranging from 0 to 44 points. The Spanish Validated Version of the MRS questionnaire was used and filled in for the purposes of this intervention trial at the four time points (baseline, and 1.5, 3, and 6 months [27].

#### 2.4.3. Anthropometric Measurements

Anthropometric measurements were obtained at the beginning and end of the trial intervention period (visit at 6 months). Resting supine 12-lead electrocardiograms were recorded at baseline.

Diastolic and systolic blood pressure (DBP and SBP) and heart rate were measured in triplicate in resting and fasting conditions using a validated semiautomatic oscillometer (Omron HEM-705CP). Trained registered staff following anthropometric standardization protocols premeasured body weight, height, and waist circumference. Participants were weighed wearing light clothing and without shoes, using a high-quality calibrated scale. Height was measured with a wall-mounted stadiometer. Waist circumference was measured at the midpoint between the lower margin of the last palpable rib and the top of the iliac crest [29]. Body mass index (kg/m^2^) (BMI) was calculated as weight (kg) divided by the height squared (m^2^).

#### 2.4.4. Biological Samples and Biochemical Analyses

Overnight fasting blood samples were collected at baseline and 1.5, 3, and 6 months of intervention. Automated biochemical profiles were measured at the Biomedical Diagnostic Center of the Hospital Clinic (Table 2).

Blood from each visit was drawn into ethylenediaminetetraacetic acid (EDTA) collection tubes, and plasma was separated after centrifugation at 1500 g (RCF) for 15 min at 7 °C. 24-h urine samples were also collected at all visits. Plasma and 24-h urine samples were stored in aliquots at −80 °C until the day of analysis.

Stored plasma aliquots collected at the different time points were used to analyse sex hormones. FSH, LH, progesterone, E2, and sex hormone-binding globulin (SHBG) were measured by a chemiluminescent immunoassay using an Atellica instrument (Siemens), while total testosterone (T-total) was measured by a direct chemiluminescent immunoassay with a Cobas instrument (Roche). The free testosterone index (FTI) was defined as the ratio between testosterone levels and SHBG levels, multiplied by a constant. To study the bioavailable E2, the free oestradiol index (FEI) was calculated as the molar ratio of plasma E2 to the plasma SHBG level and multiplying by 100 [30]. The lower detection limits of plasma E2 and progesterone were 12 pg/mL and 0.21 ng/mL, respectively; levels below these limits were defined as 11 pg/mL of E2 and 0.20 ng/mL of progesterone.

#### 2.4.5. Dietary Intake and Physical Activity Assessments

Dietary intake was recorded at baseline using a 151-item semi-quantitative food frequency questionnaire (FFQ) [31]. The total energy intake (kcal/day) and absolute consumption values of phytoestrogen-rich food subclasses (legumes, seeds, and whole grains) per day were estimated according to Spanish food composition tables and the Phenol-Explorer database [32]. Isoflavonoid intake (mg/day) was estimated from the consumption of plant-based milks, alcoholic beer, and non-alcoholic beer reported in the FFQ, multiplying the isoflavonoid content in food (mg/100g of food) by the daily consumption of each food (g/day) [31]. In addition, the 14-point Mediterranean Diet Adherence questionnaire was used as a healthy dietary index in order to evaluate differences between study groups at baseline [33]. The subjects also filled out a 7-day food record questionnaire at baseline and at the end of the intervention. These dietary registers were carefully checked and three representative days (two in the week and one at the weekend) were fully evaluated using nutrition analysis software, PCN Pro, developed at the University of Barcelona (Programa de Càlcul Nutricional Professional, Santa Coloma de Gramenet, Barcelona, Spain). Physical activity was registered at the beginning and end of the study using the Minnesota leisure-time questionnaire, previously validated in a population of Spanish women [34].

### 2.5. Statistical Analyses

Continuous variables are expressed as mean ±SD. Categorical variables are expressed as number (n) and proportion (%). Differences in the characteristics of volunteers between groups at baseline were tested by the chi-square test for categorical variables and the Kruskal–Wallis test with post-hoc Dunn’s test for continuous variables.

The effect of beer interventions on climacteric symptoms, sex hormone profile, and CVRF was estimated by performing a generalized estimating equation (GEE) on Poisson regression models for repeated measures. Identity link function, independent correlation, and robust standard error parameters were specified due to the low number of clusters and the nature of the variables [35]. Adjusted differences and their corresponding 95% CI were computed using the increasing complexity models. Climacteric symptom models included time since the onset of menopause, the baseline exposure variable (stress/depression from daily life (score 1–5), FSH levels (continuous), and isoflavonoid consumption (continuous). Sex hormone profile models included the variables described above, without taking into consideration FSH levels. An interaction term of time-exposure allowed the evaluation of potential differences between exposure intervention groups in response to changes over time.

Intragroup differences in MRS questionnaire items were assessed by a Kruskal–Wallis test followed by post-hoc Dunn’s test. Differences between baseline and 6 months in sex hormone levels and dietary patterns were analyzed by a non-parametric test for two related samples in each arm/group. A Wilcoxon matched-pair signed-rank test for small samples was applied to symmetric variables, while for asymmetric variables the sign test of matched pairs was used. Symmetry was studied by the skewness and kurtosis test for normality (control and AB group) or graphically (NAB group).

Percent changes (% changes) of hormone levels were calculated by dividing the differences between the final and the initial hormone values by the initial hormone concentration per 100. Spearman’s correlation coefficient was estimated to study linear associations between different baseline hormone levels, different % changes in hormone levels, and between individual % changes and their corresponding baseline hormone concentration. Due to their theoretical relationship, associations between individual % changes in FTI and T-total or SHBG were not studied, nor in the FEI and E2 or SHBG. Correlation coefficient values were interpreted by Chan’s guideline on the strength of the linear relationship [36].

All statistical analyses were conducted using the Stata statistical software package version 16.0 (StataCorp, College Station, TX, USA). Statistical tests were two-sided and *p*-values below 0.05 were considered significant.

## 3. Results

### 3.1. Study Subjects, Intervention, and Compliance

**Figure 1:**
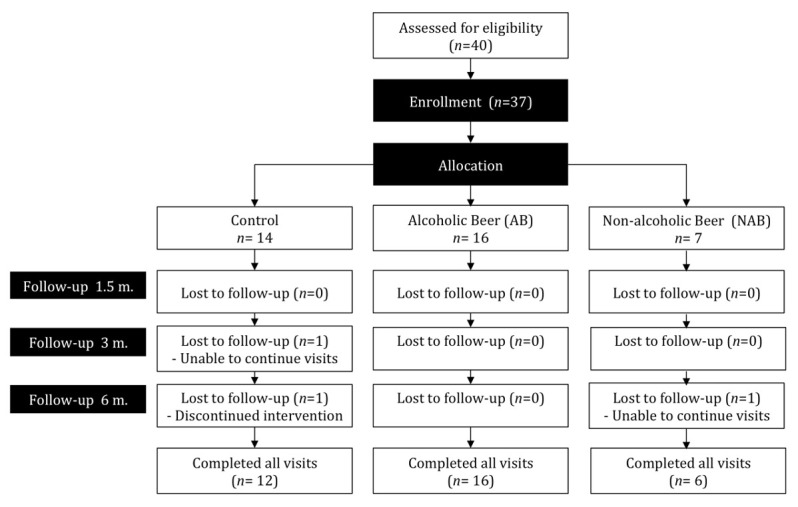
Flow diagram of participant recruitment and compliance in each phase of the intervention trial.

The recruitment and compliance of the study participants are detailed in a flow diagram (Figure 1). A total of 37 postmenopausal women were enrolled in the clinical trial and assigned to the three study groups: 16 chose the AB intervention, while 7 and 14 chose to be allocated to the NAB or the control group, respectively. Only 3 women dropped out and 34 participants finished the trial. As shown in Figure 1, two subjects from the control group and one from the NAB group dropped out due to difficulty in continuing visits or in complying with the intervention.

The prenylflavonoid contents of beers given to the AB and NAB groups are shown in Table 1. Subject compliance with the intervention was 100% according to dietary self-records at 6 months and the interviews in the different visits during the intervention. To confirm intervention compliance, IX concentrations were measured in the 24-h urine provided by the participants at all four visits. IX concentration was below the detection level <0.04 ppb at baseline [26], and increased in 93.5% of collected urine samples from both intervention groups.

### 3.2. Participant Characteristics at Baseline

The baseline characteristics of the participants are summarized in Table 2 and Table 3. The age range of the participants was 49–66 years. Study groups revealed no significant differences in terms of age, smoking habits, sleeping hours, stress/depression from work, and time since the onset of menopause. The alcoholic drinking habit and stress/depression from daily life scores were the two baseline items that were significantly different between study arms. Women in the AB group drank alcoholic beverages more frequently and were more stressed than women from the other groups. Wine and beer were the two most preferred types of alcoholic beverages in all the study groups.

Most of the baseline anthropometric measurements and biochemical parameters of the three groups were balanced, as shown in Table 2. Specifically, the study arms were similar in body weight, BMI, blood pressure (BP), lipid and thyroid profiles, and other baseline clinical characteristics. Most participants were normoweight or overweight with an elevated waist circumference, and had normal BP, heart rate, and analytical values. However, women in the AB group had significantly higher levels of FSH and aspartate transaminase compared to the other study groups.

#### Covariates

For a more in-depth study of the intervention effects, differences at baseline and changes in dietary habits, related medication history and physical activity during the intervention, were evaluated between the study groups. No statistical differences in medication use and physical activity were observed at baseline or at the end of the intervention.

Food, nutrient, and energy intakes were derived from both food records and FFQs. At baseline, significant differences in fiber intake (*p*-value: 0.008) from food recalls were found between groups (Supplementary Table S1), whereas recorded information from the FFQs did not reveal any significant differences. All dietary parameters except for polyunsaturated fatty acids studied by food records correlated significantly with the FFQs (coefficients of correlation > 4000) (*data not shown*). Before the run-in period, alcohol consumption was significantly higher among women allocated to the AB group (Control: 1.9 ± 2.4 g/day; AB: 6.6 ± 4.1 g/day; NAB: 1.9 ± 2.1 g/day; *p*-value: <0.001). The analyses of macronutrients at baseline and the end of the study revealed a low percentage of carbohydrate intake (<45–60% kcal/day), and a high percentage of total fat (>20–35% kcal/day) and saturated fatty acid (>10% kcal/day) intakes [37], without differences between study groups.

Individual changes during the study were also monitored. According to the 3-day food records, the control and NAB groups did not change their dietary habits. Furthermore, in the AB group only alcohol consumption was significantly higher in comparison to the washout period (*p*-value: <0.001)

### 3.3. Intervention Effects on Climacteric Symptoms

**Figure 2:**
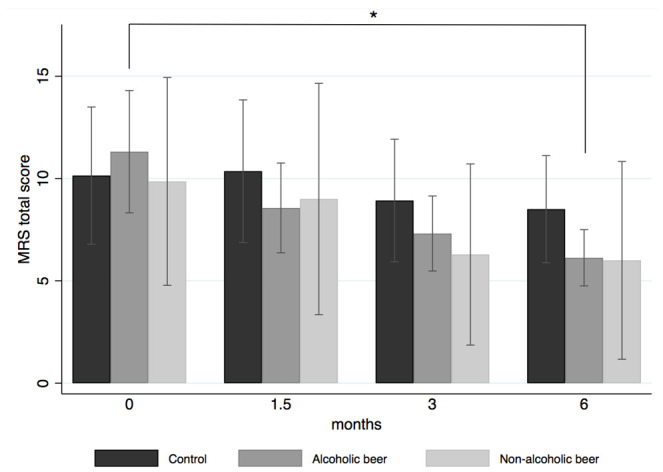
Evolution of total MRS score of the study groups during the intervention. Results are expressed as mean ± standard deviation. Means with (*) are significantly different. *p*-value <0.05.

The MRS questionnaire was used to determine the effect of AB and NAB consumption on the severity of climacteric symptoms. Before the intervention, the mean scores of the total recorded symptoms did not significantly differ between the three study arms (Figure 2).

The three most frequently experienced symptoms of the eleven composing the MRS were joint and muscular discomfort (70.3%), physical and mental exhaustion (70.3%), and sleep problems (64.9%). The mean scores for the eleven symptoms in all the study groups were between “absent” and “moderate” (0–2 points). However, as shown in Supplementary Table S2, the only significant change at 3 months in the control group was a diminished heart discomfort symptom (*p*-value: 0.028). Furthermore, women in the AB group reported a significant decrease in the total MRS score at the end of the follow-up. The NAB group did not show any statistically significant changes throughout the intervention period, although the MRS total score had a decreasing tendency of 4.2 ± 3.0. Over the intervention period, menopausal symptoms in the AB and NAB groups decreased in severity by an average of 46.0% and 42.4%, respectively, in comparison with 10.9% in the control group (Figure 2). While these results point to a positive effect of the interventions compared to the control, intra-individual differences in MRS scores as well as the time-exposure interaction provide a more precise indication of the intervention effect. 

Table 4 shows the intervention effect on MRS subscales and total MRS scores during follow-up. Menopausal women that received AB and NAB experienced a significant reduction in climacteric symptoms in comparison with those in the control group at 6 months of the intervention. The time-exposure interaction measured linearly was found to be statistically significant when comparing the AB and control groups (*p*-trend: 0.011), consisting of an expected decrease of the adjusted differences in the total MRS score between the AB and control groups of -0.6 points (95% IC: −1.1, −0.1) for each three additional months of intervention (*data not shown*). Furthermore, mild psychological symptoms (depressive mood, irritability, anxiety, and physical and mental exhaustion) after 6 months of daily moderate beer consumption decreased significantly (adjusted difference: −2.1; 95% IC: −3.5, −0.6) compared to the control group, with a significant linear time–exposure interaction (adjusted difference: −0.3; 95% IC: −0.5, −0.1; *p*-trend: 0.010). However, there were no significant differences between groups in the urogenital and somatic domains after the 6-month treatment period. The alcoholic fraction derived from AB consumption did not entail a lower or increased climacteric symptom severity (AB vs. NAB group, Table 4).

### 3.4. Intervention Effects on Sex Hormone Profile

The hormone levels at baseline and after 6 months of intervention are shown in Supplementary Table S3. A significant change in FSH levels was found in the AB group (*p*-value: 0.038). Plasma levels of LH, E2, progesterone, T-total, FTI and SHBG did not change significantly during the study in any group, nor did the FEI or the T/E2 ratio.

The intervention effect on the sex hormone profile at follow-up can be seen in Table 5. The NAB intervention resulted in a significant decrease in the FTI (adjusted difference: −0.43; 95% IC: −0.86, −0.01; *p*-value: 0.046) compared to the control group. Furthermore, the reduction in FSH (adjusted difference: −10.01; 95% IC: −14.76, −1.36; *p*-value: 0.023), and LH (adjusted difference: −4.74; 95% IC: −8.92, −0.56; *p*-value: 0.026) values was significantly higher in the AB group in comparison with the NAB and control groups, respectively. SHBG levels in both AB and NAB groups were lower at the end of the intervention period, but not significantly.

To better appreciate the changing patterns in sex hormones and the influence of the interventions on the individual hormones, each hormone value was expressed as the % change in hormone concentration, and associations between these responses were studied. In general, % changes of LH and FSH were close to being significantly correlated (r: 0.327; *p*-value: 0.059). Maintaining the inverse correlation shown at baseline, the higher the % change in E2, the lower the % change in FSH (r: −0.360; *p*-value: < 0.037). Other significant correlations found between changes in individual hormones were the % changes of FSH values and the baseline levels of this hormone (r: −0.414, *p*-value: 0.015). Interestingly, participants who initially had a higher amount of SHBG were those who had a lower increment or even a decrease of this protein at the end of the study (r: −0.591, *p*-value: < 0.001). Indeed, individuals who had SHBG values above 80 nMol/L at baseline had lower levels at 6 months, regardless of their group (*n* = 7).

### 3.5. Intervention Effects on CVRF

Changes in anthropometric and clinical variables were explored and the intervention effects on CVRF are shown in Table 6. Only mean aspartate transaminase levels differed between the control and AB groups at baseline, but all values fell within the normal range established for this enzyme (Table 2). Daily moderate AB and NAB consumption did not affect anthropometric variables after 6 months. However, DBP was found to diminish in the NAB group in comparison with the AB group (adjusted difference: −7.7; 95% IC: −13.3, −2.1; *p*-value: 0.007). Regarding the lipid profile, the beer interventions had a positive impact and reduced low-density lipoprotein cholesterol (LDL-C) levels. In this respect, it is worth mentioning that both AB and NAB groups started with higher mean levels that exceeded the reference limit. Apoliprotein A1 (ApoA1) levels decreased significantly in the NAB compared with the AB group (adjusted difference: −20.9; 95% IC: −36.6, −5.1; *p*-value: 0.010) and almost significantly with regard to the control group (adjusted difference: −16.6; 95% IC: −33.3, 0.29; *p*-value: 0.054). As for the liver profile, gamma-glutamyl transferase levels were significantly higher after both beer interventions, but final blood concentrations were still below the reference limit (Table 6).

## 4. Discussion

### 4.1. Climacteric Symptoms

Women worldwide usually find that menopausal symptoms negatively affect their quality of life. The results of this study show that a moderate beer consumption (14 g of ethanol a day) significantly reduces several menopause-related symptoms and should therefore improve the quality of life of postmenopausal women. As these improvements were observed after both AB and NAB consumption, they can be attributed to the non-alcoholic fraction of beer, possibly to the phytoestrogenic effect of polyphenols. All the variables controlled in the study have already been described as modifying factors of menopausal symptomatology [38].

The women in the study who were administered beer were consuming a daily dose of 359 ± 17.4 μg and 259 ± 10.3 μg of prenylflavonoids in the AB and NAB groups, respectively. The estrogenic effect of 8-PN, which has a higher affinity for the estrogen receptor α than β, has already been demonstrated. The relative potency of 8-PN is almost equal to that of estrone and is 70 times weaker than that of E2 [39]. In fact, the activity of 8-PN in beer is greater than the effects of phytoestrogens typically found in soya products [20,21]. Three random controlled trials analyzed the effects of 8-PN on vasomotor symptoms and other menopausal discomforts, concluding that a daily dose of 100 μg/day of 8-PN may provide relief for vasomotor symptoms after 4-12 weeks [13,14,18]. A marginal reducing effect on menopausal complaints in the MRS was also found after 8 weeks of standardized hop extract administration [13]. Therefore, as our results indicate, after the isomerization of XN into IX during brewing and subsequent metabolism of 8-PN in the human body, the effects of a marginal daily dose of phytoestrogens from beer consumption could be clinically significant [12,40].

The observed reduction of psychological symptoms after 6 months of moderate beer consumption in our intervention may also be relevant, considering their notable impact on the quality of life. No significant difference was found between the AB and NAB interventions in terms of effects on these symptoms.

In line with beer’s phytoestrogenic effect, other foods have been described as phytoestrogen sources [10,11,41]. Pomegranate seeds are rich in phytosterols [42,43], while legumes (e.g., soy, bean, alfalfa) are rich in isoflavones [10] and flaxseeds in lignans [44,45]. Due to the difference in components, doses, and duration of the interventions, as well as the variability in the metabolism among individuals and a consistent high placebo response rate; additional studies are warranted to further elucidate the association and comparison between phytoestrogen food sources and the relief of climacteric symptoms [41].

### 4.2. Sex Hormone Profile

The sex hormone profile did not differ significantly between the study groups. Substantial association with lifestyle (e.g., BMI, smoking, diet, physical activity) and physiological factors (e.g., age, time since the onset of menopause) could account for the absence of clear hormonal differences [46,47,48]. Reporting similar results, Sierksma et al. (2004) did not detect any differences in plasma E2 and T-total in postmenopausal women after a 3-week crossover random controlled trial comparing AB (30 g alcohol/day) and NAB consumption [49]. Other studies have observed lower levels of LH and FSH, and higher levels of SHBG after 4 weeks of beer consumption [50] and a 16.7% decrease in LH concentration (95% IC 0.5, 30.2) 24 h after the administration of a single 750 mg dose of 8-PN [51]. These findings suggest that 8-PN, ingested either in isolation or in beer, may be able to cross the blood–brain barrier and interact with the hypothalamic-pituitary axis [50,51].

Evidence for longitudinal changes in reproductive hormones during natural menopause transitions has been recently reviewed [47]. Menopause is characterized by a reduced synthesis and secretion of E2 by the ovaries, whereas levels of LH and FSH, the products of gonadotropin cells that can be secreted in tandem, increase for up to 5 and 7 years after the onset of menopause, respectively [47]. Postmenopausal estrogens are synthesized from androgens derived from the metabolism of estrone [52], and the release of pituitary-ovarian hormones is controlled by a negative feedback system [3,46,53]. Thus, the inverse correlation found between E2 and FSH at baseline and after the intervention suggests that E2 still affects pituitary FSH output during the postmenopausal state and continues to play an important role in FSH control. As in other studies, the mean levels of FSH in our volunteers were around 2-fold higher than LH, and both hormone values were directly correlated [46,47].

SHBG decreases slightly for about 4 years after the onset of menopause, after which it increases to a small extent [47]. The bioavailability of both E2 and T-total depends on the fractions that are free or transported with albumin in the circulation, as these have rapid access to target tissues, unlike the fraction bound to SHBG [54]. In the present study, changes in SHBG and the bioavailability of E2 and T-total were explored. T-total seemed to decrease after 6 months of NAB consumption due to a reduction in the FTI. Despite this decrease and the reduction in SHBG levels, T-total bioavailability was apparently not affected by the AB intervention and only slightly by the NAB intervention. As the bioavailability of E2 was also stable, the estrogenic effect of beer consumption cannot be explained by lower SHBG levels.

The FSH levels in the AB group decreased significantly more than in the NAB group after the interventions, despite higher baseline values. Our findings are in line with Soares et al. (2020) [47], who conclude that changes in sex hormones do not differ between alcohol drinkers and abstainers, but that females who drink alcohol more often have higher FSH levels from 2 years after menopause and lower SHBG levels throughout the reproductive age than those with a lower alcohol intake frequency [47]. A decrease in free-E2 and free-T and an increase in SHBG has also been related with a loss of total body fat [4].

### 4.3. Cardiovascular Risk Factors

The analysis of anthropometric and biochemical parameters revealed the safety of a daily moderate AB and NAB consumption and the plausible role of NAB in the management of the lipid profile and BP in postmenopausal women. Indeed, the effect of moderate alcohol consumption on CVRF in a controlled crossover dietary study was found to be significantly higher in postmenopausal than premenopausal women [55].

#### 4.3.1. Body Weight and Fat

The incidence of most cardiovascular diseases in women increases after menopause when estrogen levels decrease [52]. Evolution in body weight and body fat distribution in either of the interventions did not differ significantly compared to the control group, which does not offer conclusive proof of a beneficial or negative effect of moderate AB and NAB consumption on these health parameters. The results obtained after the 6-month intervention are in line with the available literature, which indicates that beer consumption has an inconsistent effect on adiposity and weight-control outcomes in women [56].

#### 4.3.2. Blood Pressure

No significant effect on BP was observed after AB consumption compared to the control group, but a significant decrease was seen in the women who consumed NAB compared to those in the AB group. This difference between the two drinks may be due to the pressor effects of ethanol, which could counteract the vasodilator properties attributed to polyphenols [57]. The effect of both phytoestrogens and alcohol on BP has been studied [57,58,59,60,61,62]. Husain et al. (2015) observed no significant change but a reducing trend in SBP or DBP in postmenopausal women after an intervention with soy isoflavones [58]. Another study showed that isoflavone intake reduces SBP and that the consumption of soy foods tends to reduce both SBP and DBP [61]. Moreover, a reduction in SBP was found after NAB intake (990 mL/day) for 4 weeks in 61 ± 6 year-old men, while DBP remained unchanged. AB (660 mL/day) and gin (30g/day) consumption did not show any effect on BP [57].

#### 4.3.3. Lipid Profile

A decrease in LDL-C levels was observed after NAB intake for 6 months. However, AB did not seem to affect the lipid profile, in contrast with another study that related ethanol consumption to an increase in total cholesterol, HDL-C and ApoA1 [62]. Interestingly, Chiva et al. (2015) observed lower ApoA1 levels (≈0.5%) after NAB intake but higher HDL-C, ApoA1 and adiponectin after moderate gin and AB consumption [57]. Thus, the non-alcoholic and alcohol fractions of beer did not exhibit the same beneficial effects. A similar study on healthy postmenopausal women reported no differential effect on HDL-C and ApoA1 after moderate consumption of AB or NAB after 3 weeks [63].

The impact of alcohol on lipoproteins in postmenopausal women receiving a controlled diet for 8 weeks differed according to the dose: after the intake of 15 g/day of alcohol, LDL-C and triglycerides significantly decreased, while the benefit of an increase in HDL-C and ApoA1 was only significant after 30 g/day of alcohol [55]. In our AB group, women consumed only 14 g of alcohol daily, an insufficient dose to observe an effect on HDL-C.

Although the results of previous studies suggest a leading role of alcohol in the health effects of beer, some of the study designs have not taken into account that NAB has a lower amount of polyphenols than AB. Hence, the overall impact and mechanisms of action of beer polyphenols might not have been well elucidated yet [62].

#### 4.3.4. Hepatic Profile

The liver plays an important role in the enterohepatic recycling of cholesterol and other substances. Higher levels of aspartate transaminase, alanine transaminase, and gamma glutamyl transpetidase (GGT) are related with hepatocyte damage. In this study, both beer interventions increased GGT levels after 6 months. In a previous study, the phytoestrogens tested were not significantly associated with changes in GGT concentration, although a notable negative association between enterolactone, a well-known phytoestrogen metabolite, and GGT levels in urine was reported [64]. The lack of evidence on this relationship hinders the interpretation of the present results.

### 4.4. Strengths and Limitations

To our knowledge, this is the first human trial specifically conducted to investigate the effect of beer, with or without ethanol, on the menopausal transition in healthy postmenopausal women. The proposed level of phytoestrogen intake was limited in order to comply with the current dietary Spanish guidelines for alcohol intake, which recommend a maximum of 140 g/week of alcohol for women who are habitual drinkers [65]. The NAB intervention was designed to provide a similar amount of total phytoestrogens as regular beer, this being one of the major strengths of this study. However, a dose-response relationship between prenylflavonoids from beer and menopausal symptoms remains undetermined.

An umbrella systematic review and meta-analyses published in 2007 stated that intervention studies with phytoestrogens without specific inclusion criteria might underestimate the clinical efficacy of this therapeutic approach to menopausal symptoms. Thus, the menopausal status (age and time since the onset of menopause), the description of the intervention (type and amount of phytoestrogen), and the baseline intensity of symptoms are key factors in this kind of study. It was concluded that phytoestrogens could be used in early menopausal women (<5 years since menopause) with mild to moderate vasomotor symptoms [8]. In our clinical trial, the participant population was suitable to study the intervention effect on menopausal complaints, as the age range was narrowed to 49–66 years and the mean times since the onset of menopause in the three study arms were 52.1, 36.7 and 46.0 months (3–4.3 years). Moreover, the phytoestrogen intervention was well-characterized, and the severity of the described symptoms was mild to moderate at baseline. As menopausal complaints naturally decrease over longer time periods, 6 months of follow-up seems to be an appropriate timeframe [13]. Additional strengths of the present study are that drinkers were consuming a single type of alcoholic beverage throughout, and good intervention compliance was achieved.

Although our findings indicate that the beer interventions produced significant differences in comparison with the control group, the tentatively positive effect should be interpreted with caution. The greatest weakness of the present trial is the small sample size, which may have insufficient statistical power to identify some of the effects (power AB vs. Control = 65%; power NAB vs. Control = 34%). Nevertheless, statistically significant differences were observed between the two beer interventions and the control group, pointing to a clinically relevant effect. Moreover, participants were not randomized, but reflected real life conditions. Other limitations include the intra-individual variability of the exposure effect, and a probable self-selection bias, as participants were recruited by an advertisement and volunteered to participate in this clinical trial.

## 5. Conclusions

In conclusion, a daily moderate AB and NAB consumption may provide an alternative approach for postmenopausal women seeking relief from mild to moderate climacteric symptoms. Moreover, NAB was found to have a beneficial effect on LDL-C, ApoA1, and DBP measurements, all known risk factors for cardiovascular disease. However, these results must be considered as preliminary and will require confirmation with larger sample sizes.

The clinical implications of daily moderate AB and NAB consumption have been revealed in this study, but the mechanisms of action and impacts on sex hormones remain unknown. The most effective quantity of beer, with or without alcohol, that can be safely consumed by a postmenopausal woman still needs to be determined, taking into consideration factors such as age, genetics, and ethnicity.

## Figures and Tables

**Table 1:** Intervention-phytoestrogen content of the administered alcoholic (AB) and non-alcoholic beer (NAB).

Intervention Group	IX μg/Day	XN μg/Day	8-PN μg/Day	6-PN μg/Day	Total Amount μg/Day	Alcohol g/Day
AB	302.7 ± 16.8	27.9 ± 0.6	5.5 ± 0.4	22.8 ± 0.3	358.9 ± 17.4	14
NAB	104.7 ± 3.8	81.3 ± 4.0	10.3 ± 0.8	62.7 ± 2.2	259.0 ± 10.3	0.0

6-PN, 6-prenylnaringenin; 8-PN, 8-prenylnaringenin; IX, isoxanthohumol; XN, xanthohumol. Values are means of three analyses ± SDs.

**Table 2:** Baseline anthropometric measurements and biochemical analysis according to the intervention group.

	Control (*n* = 14)	AB (*n* = 16)	NAB (*n* = 7)	*p*-Value
Weight, kg	71.7 ± 13.0	64.7 ± 10.3	75.2 ± 20.3	0.324
BMI, kg/m^2^	27.2 ± 4.4	25.3 ± 3.7	30.0 ± 9.0	0.634
WC, cm	89.4 ± 9.7	87.0 ± 10.3	90.6 ± 16.8	0.810
DBP, mmHg	74 ± 13	73 ± 6	74 ± 6	0.944
SBP, mmHg	121 ± 15	120 ± 14	120 ± 16	0.929
Heart rate, bpm	70 ± 12	68 ± 11	71 ± 7	0.657
Glucose, mg/dL	90.9 ± 6.2	93.9 ± 7.7	97.1 ± 11.5	0.376
Creatinine, mg/dL	0.69 ± 0.12	0.64 ± 0.10	0.70 ± 0.11	0.483
Uric acid, mg/dL	4.9 ± 0.7	4.6 ± 1.3	5.3 ± 1.7	0.322
Total cholesterol, mg/dL	185 ± 30	206 ± 22	208 ± 27	0.060
LDL-C, mg/dL	114 ± 23	135 ± 25	142 ± 18	0.063
HDL-C, mg/dL	56 ± 13	57 ± 8	56 ± 13	0.895
Triglycerides, mg/dL	69 ± 32	77 ± 25	66 ± 18	0.663
ApoA1, mg/dL	150 ± 19	163 ± 14	158 ± 22	0.058
ApoB mg/dL	94 ± 17	106 ± 20	105 ± 15	0.188
AST, U/L	21 ± 12 ^a^	26 ± 10 ^b^	22 ± 5 ^ab^	0.025
ALT, U/L	19 ± 10	24 ± 14	18 ± 5	0.217
GGT, U/L	14 ± 5	22 ± 10	20 ± 15	0.057
Total proteins, g/L	70 ± 3	69 ± 5	71 ± 4	0.541
Albumin, g/L	42 ± 2	43 ± 3	42 ± 2	0.530
TSH, ng/mL	2.11 ± 1.50	2.63 ± 2.23	2.58 ± 0.50	0.290
FT4, ng/mL	1.16 ± 0.14	1.15 ± 0.16	1.07 ±0.07	0.318
T3, ng/mL	1.16 ± 0.21	1.15 ± 0.15	1.24 ± 0.40	0.961
Cortisol, μg/dL	13.3 ± 4.1	13.7 ± 5.1	14.2 ± 5.5	0.900
PTH, ng/mL	65.3 ± 24.2	61.6 ± 21.9	66.0 ± 17.8	0.653
25-hydroxy-vitamin D3, ng/mL	23.2 ± 4.8	27.4 ± 10.5	24.8 ± 13.5	0.656
FSH, U/L	66.7 ± 21.5 ^a^	103.0 ± 44.4 ^b^	55.8 ± 22.5 ^a^	0.006
E2, pg/mL	24.1 ± 12.8	19.9 ± 8.1	22.5 ± 7.7	0.651

AB: alcoholic beer; ALT: alanine transaminase; ApoA1: Apolipoprotein A1; ApoB: Apolipoprotein B; AST: aspartate transaminase; BMI: body mass index; DBP: diastolic blood pressure; E2: 17-β-estradiol; FSH: follicle-stimulating hormone; FT4: thyroxine; GGT: gamma-glutamyl transferase; HDL-C: high-density lipoprotein cholesterol; LDL-C: low-density lipoprotein cholesterol; NAB: non-alcoholic beer; PTH: parathyroid hormone; SBP: systolic blood pressure; T3: tri-iodothyronine; TSH: thyroid stimulating hormone; WC: waist circumference. Results are presented as mean ± THE. Kruskal Wallis test followed by post-hoc Dunn’s test was used for statistical comparisons. Means within the same row carrying different superscripts (^a,b^) are significantly different. *p*-value < 0.05.

**Table 3:** Baseline characteristics of the participants according to intervention group.

	Control (*n =* 14)	AB (*n =* 16)	NAB (*n =* 7)	*p*-Value
**Medical records**				
Age, *years*	55.6 ± 5.1	54.9 ± 3.6	56.4 ± 3.2	0.647
Smoking habit				
*Current*, *n (%)*	1 (7.1)	6 (37.5)	2 (28.6)	0.170
*Former*, *n (%)*	3 (21.4)	4 (25.0)	0 (0.0)	
*Never*, *n (%)*	10 (71.4)	6 (37.5)	5 (71.4)	
Sleeping time, *hours*	6.4 ± 0.9	7.0 ± 1.3	6.8 ± 1.0	0.448
Stress/depression from daily life^1^	2.6 ± 1.4^ab^	3.4 ± 1.0^a^	1.7± 1.1^b^	0.013
Stress/depression from work^1^	2.9 ± 1.5	2.7 ± 1.6	2.6 ± 1.3	0.881
Time since the onset of menopause, *months*	52.1 ± 35.5	36.7 ± 28.0	46.0 ± 55.5	0.432
Physical activity, *MET*-*min/day*	731 ± 449	681 ± 616	467 ± 118	0.587
**Dietary history**				
Total energy intake, *kcal/day*	2695 ± 517	2726 ± 673	2352 ± 264	0.189
MedDiet, *14*-*item score*	8.7 ± 1.8	7.4 ± 1.6	7.9 ± 2.4	0.170
Legumes, *g/day*	62 ± 31	53 ± 23	43 ± 24	0.586
Seeds, *g/day*	5.0 ± 8.4	0.9 ± 1.5	1.7 ± 1.9	0.208
Whole grains, g/day	51 ± 39	95 ± 83	72 ± 69	0.419
Isoflavonoids, *mg/day*	6.4 ± 13.2	2.3 ± 5.3	10.3 ± 17.6	0.079
Alcohol drinking habit				
*Weekly*, *n (%)*	1 (7.1)	9 (56.3)	1 (14.3)	0.025
*Occasionally*, *n (%)*	10 (71.4)	7 (43.8)	5 (71.4)	
*Never*, *n (%)*	3 (21.4)	0 (0.00)	1 (14.3)	
Type of alcohol preferred				
*Beer*, *n (%)*	3 (21.4)	8 (50.0)	3 (42.9)	0.482
*Wine*, *n (%)*	7 (50.0)	7 (43.8)	3 (42.9)	
*Spirits*, *n (%)*	1 (7.1)	1 (6.3)	0 (0.0)	
*None*, *n (%)*	3 (21.4)	0 (0.0)	1 (14.3)	
**Medication**				
Antihypertensive agents, *n (%)*	1 (7.1)	3 (18.7)	1 (14.3)	0.649
Lipid-lowering medication, *n (%)*	0 (0.0)	2 (12.5)	0 (0.0)	0.250
Antidepressants, sedatives, anxiety pills, *n (%)*	3 (21.4)	3 (18.8)	0 (0.0)	0.425
Sleeping pills, *n (%)*	2 (14.3)	2 (12.5)	0 (0.0)	0.585
Dietary supplements, *n (%)*	3 (21.4)	8 (50.0)	4 (57.1)	0.172

^1^ score from 1–5. AB: alcoholic beer; MedDiet: Mediterranean Diet Adherence Screener 14-item score; NAB: non-alcoholic beer. Continuous variables are presented as mean ± SD. Categorical variables are expressed as number (n) and proportion (%). Chi-square test was applied to study differences in categorical variables. Kruskal–Wallis test followed by post-hoc Dunn’s test was applied to study differences in continuous variables. Means within the same row carrying different superscripts ^(a, b)^ are significantly different. *p*-value < 0.05.

**Table 4:** Intervention effect on somatic, psychological, and urogenital domain scores and total MRS (Menopausal Rating Scale) score at follow-up.

	AB vs. Control			NAB vs. Control			AB vs. NAB		
	Difference Time-Exposure (95% IC)	*p*-Value	*p*-Trend	Difference Time-Exposure (95% IC)	*p*-Value	*p*-Trend	Difference Time-Exposure (95% IC)	*p*-Value	*p*-Trend
Somatic Model 1 Model 2 Model 3	−1.1 (−2.7, 0.6) −1.2 (−2.7, 0.3) −1.3 (−2.8, 0.2)	0.199 0.120 0.088	0.223 0.126 0.083	−1.0 (−3.2, 1.2) −1.5 (−3.7, 0.7) −1.7 (−4.0, 0.5)	0.354 0.184 0.128	0.367 0.230 0.138	−0.0 (−1.9, 1.0) 0.3 (−1.7, 2.2) 0.5 (−1.6, 2.5)	0.988 0.779 0.665	0.993 0.890 0.737
Psychological Model 1 Model 2 Model 3	−2.1 (−3.8, −0.5) −2.1 (−3.5, −0.7) −2.1 (−3.5, −0.6)	0.011 0.004 0.004	0.021 0.007 0.010	−1.1 (−2.8, 0.6) −1.1 (−2.8, 0.7) −1.3 (−2.9, 0.3)	0.196 0.236 0.120	0.254 0.321 0.157	−1.0 (−2.9, 0.9) −1.0 (−3.1, 1.0) −0.8 (−2.6, 1.0)	0.290 0.313 0.396	0.377 0.331 0.415
Urogenital Model 1 Model 2 Model 3	−0.4 (−1.3, 0.5) −0.4 (−1.3, 0.4) −0.5 (−1.4, 0.3)	0.367 0.309 0.214	0.354 0.252 0.170	−0.1 (−1.2, 1.0) −0.5 (−1.7, 0.7) −0.7 (−1.9, 9.5)	0.861 0.397 0.236	0.898 0.459 0.288	−0.3 (−1.3, 0.7) 0.1 (−1.1, 1.2) 0.2 (−1.0, 1.3)	0.544 0.915 0.783	0.488 0.949 0.933
Total MRS Model 1 Model 2 Model 3	−3.5 (−6.8, −0.3) −3.8 (−6.8, −0.9) −3.9 (−6.9, −1.0)	0.031 0.011 0.009	0.041 0.013 0.011	−2.2 (−5.3, 0.9) −3.0 (−5.9, −0.1) −3.1 (−6.0, −0.2)	0.160 0.040 0.033	0.191 0.073 0.062	−1.3 (−4.2 1.6) −0.8 (−3.7, 2.2) −0.8 (−3.8, 2.2)	0.376 0.602 0.601	0.354 0.465 0.456

AB: alcoholic beer; NAB: non-alcoholic beer. Generalized estimating equation (GEE) models were used to estimate the effect (difference) of the intervention among study groups. Model 1: time since onset of menopause; Model 2: adjusted as in Model 1 plus stress/depression from daily life (score 1–5) and follicle-stimulating hormone concentration; Model 3: adjusted as in model 2 plus isoflavonoid consumption (mg/day) at baseline. *p*-value < 0.05.

**Table 5:** Intervention effect on female sex hormone profile at follow-up.

	AB vs. Control		NAB vs. Control		AB vs. NAB	
	Difference Time-Exposure (95% IC)	*p*-Value	Difference Time-Exposure (95% IC)	*p*-Value	Difference Time-Exposure (95% IC)	*p*-Value
**LH** Model 1 Model 2 Model 3	−5.02 (−9.30, −0.73) −4.77 (−9.03, −0.51) −4.74 (−8.92, −0.56)	0.022 0.028 0.026	−1.44 (−9.14, 6.27) −1.25 (−9.18, 6.68) −1.09 (−8.99, 6.80)	0.714 0.758 0.786	−3.55 (−11.26, 4.17) −3.52 (−11.35, 4.34) −3.71 (−11.68, 4.26)	0.367 0.382 0.361
**FSH** Model 1 Model 2 Model 3	−7.70 (−17.23, 1.83) −7.20 (−16.85, 2.45) −6.25 (−16.26, 3.76)	0.113 0.144 0.221	2.29 (−8.37, 12.95) 2.89 (−7.88, 13.66) 3.76 (−7.23, 14.76)	0.674 0.599 0.502	−9.99 (−18.80, −1.18) −10.09 (−19.01, −1.17) −10.01 (−14.76, −1.36)	0.026 0.027 0.023
**E2** Model 1 Model 2 Model 3	−2.61 (−17.88, 12.66) −2.32 (−17.64, 13.00) −2.22 (−17.40, 12.96)	0.738 0.766 0.774	−7.53 (−21.61, 6.55) −7.39 (−21.55, 6.76) −7.30 (−21.44, 6.84)	0.295 0.306 0.312	4.92 (−6.64, 16.47) 5.07 (−6.47, 16.61) 5.08 (−6.45, 16.60)	0.404 0.389 0.388
**Progesterone** Model 1 Model 2 Model 3	0.03 (−0.13, 0.18) 0.02 (−0.12, 0.16) 0.02 (−0.11, 0.15)	0.742 0.802 0.803	−0.00 (−0.17, 0.16) −0.01 (−0.17, 0.14) −0.02 (−0.16, 0.12)	0.984 0.861 0.822	0.03 (−0.05, 0.11) 0.03 (−0.05, 0.11) 0.03 (−0.05, 0.11)	0.502 0.446 0.414
**SHBG** Model 1 Model 2 Model 3	−10.08 (−20.77, 0.61) −10.16 (−20.87, 0.55) −10.00 (−20.82, 0.83)	0.065 0.063 0.070	−11.37 (−26.60, 3.86) −11.28 (−26.72, 4.16) −10.60 (−26.26, 5.07)	0.143 0.152 0.185	1.29 (−14.31, 16.90) 1.11 (−14.64, 16.87) 0.60 (−15.52, 16.72)	0.871 0.890 0.942
**T-total** Model 1 Model 2 Model 3	−2.91 (−8.72, 2.90) −3.08 (−8.82, 2.66) −2.70 (−8.31, 2.91)	0.327 0.292 0.345	−6.21 (−12.31, −0.11) −6.70 (−12.75, −0.66) −5.56 (−11.94, 0.82)	0.046 0.030 0.088	3.30 (−0.53, 7.13) 3.62 (−0.16, 7,41) 2.86 (−1.50, 7.21)	0.091 0.061 0.199
**TFI** Model 1 Model 2 Model 3	−0.23 (−0.71 0.24) −0.25 (−0.72, 0.21) −0.24 (−0.69, 0.22)	0.335 0.282 0.312	−0.42 (−0.86, 0.01) −0.47 (−0.90, −0.05) −0.43 (−0.86, 0.01)	0.054 0.029 0.046	0.19 (−0.14, 0.53) 0.22 (−0.08, 0.52) 0.20 (−0.10, 0.50)	0.259 0.155 0.194
**FEI** Model 1 Model 2 Model 3	0.004 (−0.113, 0.122) 0.011 (−0.109, 0.131) 0.011 (−0.108, 0.131)	0.943 0.857 0.852	−0.031 (−0.154, 0.092) −0.026 (−0.151, −0.100) −0.025 (−0.149, 0.099)	0.619 0.688 0.689	0.035 (−0.043, 0.114) 0.037 (−0.041, 0.114) 0.037 (−0.040, 0.113)	0.377 0.351 0.349

AB: alcoholic beer; FEI: free estrogen index; FSH: follicle-stimulating hormone; LH: Luteinizing hormone; NAB: non-alcoholic beer; SHBG: sex hormone-binding globulin; FTI: free testosterone index; T-Total: total testosterone. Generalized estimating equation (GEE) models were used to estimate the effect (difference) of the intervention among study groups. Model 1: time since onset of menopause; Model 2: adjusted as in Model 1 plus stress/depression from daily life (score 1-5) at baseline; Model 3: adjusted as in model 2 plus isoflavonoid consumption (mg/day) at baseline. *p*-value < 0.05.

**Table 6:** Intervention effect on cardiovascular risk factors and hepatic profile at follow-up.

	AB vs. Control		NAB vs. Control		AB vs. NAB	
	Difference Time-Exposure (95% IC)	*p*-Value	Difference Time-Exposure (95% IC)	*p*-Value	Difference Time-Exposure (95% IC)	*p*-Value
Weight, kg	−0.4 (−3.0, 2.1)	0.742	−6.0 (−16.6, 4.6)	0.267	5.6 (−4.8, 16.0)	0.293
BMI, kg/m^2^	−0.4 (−1.4, 0.7)	0.487	−2.8 (−7.1, 1.6)	0.218	2.4 (−1.9, 6.7)	0.275
WC, cm	−0.2 (−3.1, 2.7)	0.887	−5.2 (−12.3, 1.9)	0.150	5.0 (−2.0, 12.3)	0.160
DBP, mmHg	1.4 (−3.6, 6.3)	0.590	−6.3 (−12.9, 0.2)	0.057	7.7 (2.1, 13.3)	0.007
SDP, mmHg	−1.7 (−8.8, 5.4)	0.639	−10.8 (−22.5, 0.9)	0.070	9.1 (−2.2, 20.5)	0.115
Heart rate, bpm	3.6 (−2.3, 9.5)	0.233	−0.4 (−5.9, 5.1)	0.886	4.0 (−1.1, 9.1)	0.125
Glucose, mg/dL	0.7 (−3.3, 4.7)	0.735	3.1 (−5.9, 12.1)	0.496	−2.4 (−11.5, 6.7)	0.601
Total cholesterol, mg/dL	−6.0 (−19.6, 7.6)	0.386	−10.1 (−26.1, 5.8)	0.212	4.1 (−9.7, 17.9)	0.558
LDL-C, mg/dL	−12.8 (−26.4, 0.8)	0.064	−16.1 (−29.2, −3.1)	0.016	3.3 (−9.3, 15.9)	0.600
HDL-C, mg/dL	3.5 (−3.6, 10.6)	0.403	1.3 (−6.0, 8.6)	0.734	2.2 (−6.0, 10.5)	0.518
Triglycerides, mg/dL	7.2 (−11.3, 25.8)	0.446	5.3 (−12.4, 23.0)	0.558	1.9 (−14.5, 18.3)	0.817
ApoA1, mg/dL	4.4 (−13.5, 22.2)	0.633	−16.6 (−33.3, 0.29)	0.054	20.9 (5.1, 36.6)	0.010
ApoB, mg/dL	−2.3 (−12.8, 8.1)	0.663	−3.8 (−17.7, 10.2)	0.598	1.4 (−13.8, 16.7)	0.853
Lpa, mg/dL	18.1 (−6.5, 42.8)	0.149	13.4 (−11.7, 38.4)	0.295	4.8 (−5.6, 14.1)	0.319
AST, U/L	0.4 (−7.8, 8.6)	0.922	−0.9 (−8.6, 6.8)	0.821	1.3 (−5.5, 8.1)	0.706
ALT, U/L	1.2 (−5.0, 7.5)	0.705	−0.7 (−6.6, 5.2)	0.813	1.9 (−3.0, 6.9)	0.445
GGT, U/L	7.2 (0.3, 14.2)	0.042	6.4 (1−1, 11.6)	0.018	0.9 (−6.5, 8.2)	0.817

AB: alcoholic beer; ALT: alanine transaminase; ApoA1: apolipoprotein A1; ApoB: apolipoprotein B; AST: aspartate transaminase; BMI: body mass index; DBP: diastolic blood pressure; GGT: gamma-glutamyl transferase; HDL-C: high-density lipoprotein cholesterol; LDL-C: low-density lipoprotein cholesterol; Lpa: lipoprotein a; NAB: non-alcoholic beer; SDP: systolic blood pressure; WC: waist circumference. Generalized estimating equation (GEE) models were used to estimate the effect (difference) of the intervention among study groups. *p*-value < 0.05.

## Data Availability

The datasets generated during and/or analyzed during the current study are available upon request from R.M.L-R.

## Supplementary Materials

The following are available online at https://www.mdpi.com/article/10.3390/nu13072278/s1, Table S1: Baseline dietary habits of the 3-day food records from all participants in the intervention groups; Table S2: Intragroup analyses of somatic, psychological, and urogenital subscale scores and total MRS score before, during and at the end of the intervention study; Table S3: Intragroup analysis of female sex hormone levels before and after the intervention.
