# Supplementary material for: Moderate Consumption of Beer (with and without Ethanol) and Menopausal Symptoms: Results from a Parallel Clinical Trial in Postmenopausal Women
Source: Nutrients. 2021 Jun 30;13(7):2278. doi: 10.3390/nu13072278 (PMC8308431; doi:10.3390/nu13072278)
Supplement: Supplementary file 1 [file nutrients-13-02278-s001.zip › nutrients-1258324-supplementary.pdf]

Supplementary Material

Table S1. Baseline dietary habits of the 3-day food records from all participants in the intervention groups.

	Control (n= 14)	AB (n= 16)	NAB (n= 7)	p-value
Carbohydrates (% kcal/day)	38.4 ± 5.9	36.6 ± 7.5	42.1 ± 2.9	0.127
Sugar	19.3 ± 6.1	16.8 ± 4.8	21.0 ± 4.3	0.104
Protein (% kcal/day)	19.4 ± 4.0	19.1 ± 2.9	18.0 ± 2.1	0.486
Total fat (% kcal/day)	42.0 ± 7.1	43.5 ± 6.5	39.6 ± 3.6	0.611
SFA	12.1 ± 3.9	11.4 ± 3.0	10.8 ± 3.1	0.904
MUFA	20.0 ± 4.3	21.4 ± 5.0	17.4 ± 2.2	0.109
PUFA	6.6 ± 1.2	6.4 ± 1.5	7.8 ± 1.8	0.276
Fiber (g/day)	25.9 ± 9.0 a	17.0 ± 6.4 b	32.4 ± 14.5 a	**0.008**

AB: alcoholic beer; MUFA: mono-unsaturated fatty acids; NAB: non-alcoholic beer; PUFA: poly-unsaturated fatty acids; SFA: saturated fatty acids.
Kruskal-Wallis with post-hoc Dunn's test analysis was applied to study differences in continuous variables. Means within the same row carrying different superscripts (a,b) are significantly different (p-value <0.05).

Table S2. Intragroup analyses of somatic, psychological, and urogenital subscales scores and total MRS score before, during and at the end of the intervention study.

		Baseline Mean ± SD	1.5 months Changes ± SD	3 months Changes ± SD	6 months Changes ± SD	p-value
**Somatic subscale**	*Control*	3.9 ± 2.4	-0.2 ± 1.6	-0.5 ± 2.5	-0.6 ± 2.7	0.751
	AB	4.7 ± 2.8	-1.3 ± 1.5	-1.4 ± 1.8	-1.8 ± 1.7	0.277
	NAB	4.6 ± 2.9	-0.3 ± 2.2	-1.6 ± 2.9	-2.0 ± 2.1	0.590
Hot flashes, sweating	*Control*	0.9 ± 1.0	0.1 ± 0.9	0.0 ± 0.8	-0.2 ± 0.8	0.897
	AB	1.1 ± 1.2	0.0 ± 0.5	0.2 ± 0.5	-0.3 ± 0.9	0.957
	NAB	1.1 ± 1.1	0.0 ± 1.0	-0.4 ± 1.0	-0.5 ± 0.8	0.586
Heart discomfort	*Control*	0.5 ± 0.6a	-0.3 ± 0.6a	-0.4 ± 0.8b	-0.5 ± 0.7b	**0.028**
	AB	0.6 ± 0.7	-0.4 ± 0.6	-0.4 ± 0.7	-0.4 ± 0.8	0.071
	NAB	0.6 ± 0.8	0.1 ± 0.4	-0.1 ± 0.4	-0.3 ± 0.8	0.646
Sleep problems	*Control*	1.0 ± 1.1	0.2 ± 0.6	0.2 ± 0.7	0.3 ± 1.1	0.974
	AB	1.6 ± 1.5	-0.5 ± 0.9	-0.5 ± 1.0	-0.8 ± 0.9	0.480
	NAB	1.6 ± 1.3	0.0 ± 0.5	-0.3 ± 1.0	-0.5 ± 0.8	0.896
Joint and muscular discomfort	*Control*	1.4 ± 1.2	-0.3 ± 0.9	-0.4 ± 1.3	-0.3 ± 1.2	0.742
	AB	1.4 ± 1.2	-0.3 ± 0.6	-0.3 ± 0.8	-0.4 ± 1.1	0.788
	NAB	1.3 ± 1.0	-0.4 ± 0.8	-0.7 ± 1.1	-0.7 ± 0.8	0.441
**Psychological subscale**	*Control*	3.5 ± 2.8	0.0 ± 1.0	0.2 ± 1.9	-0.4 ± 1.4	0.949
	AB	4.1 ± 3.4	-1.4 ± 1.4	-2.2 ± 2.3	-2.7 ± 2.7	0.055
	NAB	3.1 ± 1.9	-0.6 ± 1.0	-1.3 ± 1.3	-1.5 ± 2.1	0.393
Depressive mood	*Control*	0.8 ± 0.7	0.1 ± 0.5	0.1 ± 0.8	-0.3 ± 0.5	0.516
	AB	1.3 ± 1.3	-0.5 ± 0.5	-0.9 ± 0.9	-1.0 ± 1.0	0.075
	NAB	1.1 ± 0.9	-0.3 ± 0.5	-0.7 ± 1.0	-0.8 ± 1.3	0.183
Irritability	*Control*	0.9 ± 1.1	0.1 ± 0.7	0.1 ± 1.0	0.1 ± 0.9	0.940
	AB	0.8 ± 1.0	-0.3 ± 0.6	-0.4 ± 0.7	-0.4 ± 0.8	0.575
	NAB	0.6 ± 0.5	-0.1 ± 0.4	-0.3 ± 0.5	0.0 ± 0.8	0.475
Anxiety	*Control*	0.7 ± 1.0	-0.1 ± 0.4	-0.1 ± 0.5	-0.3 ± 0.5	0.903
	AB	0.7 ± 1.0	-0.3 ± 0.6	-0.4 ± 0.7	-0.6 ± 0.8	0.330
	NAB	0.0 ± 0.0	0.1 ± 0.4	0.0 ± 0.0	0.0 ± 0.0	0.414
	Control	1.2 ± 0.9	0.1 ± 0.5	-0.1 ± 0.6	0.0 ± 1.0	0.896
	AB	1.3 ± 1.1	-0.3 ± 0.7	-0.5 ± 0.9	-0.7 ± 1.3	0.262

Physical and mental exhaustion	*NAB*	1.3 ± 1.1	-0.1 ± 0.4	-0.1 ± 0.4	-0.2 ± 0.4	0.959
**Urogenital subscale**	*Control*	2.8 ± 2.0	0.4 ± 1.2	-0.1 ± 1.0	-0.1 ± 1.1	0.876
	AB	2.6 ± 2.0	-0.1 ± 0.6	-0.4 ± 1.0	-0.8 ± 1.0	0.663
	NAB	2.1 ± 1.9	0.0 ± 1.2	-0.7 ± 1.1	-0.7 ± 1.2	0.893
Sexual problems	*Control*	0.6 ± 1.1	0.1 ± 0.5	0.0 ± 0.4	0.0 ± 0.4	0.876
	AB	1.1 ± 1.3	-0.1 ± 0.3	-0.1 ± 0.4	-0.2 ± 0.5	0.960
	NAB	0.6 ± 1.1	-0.1 ± 0.4	-0.1 ± 0.4	0.0 ± 0.0	0.851
Bladder problems	*Control*	0.9 ± 1.1	0.1 ± 0.7	-0.1 ± 0.9	0.0 ± 0.1	0.889
	AB	0.8 ± 0.9	-0.1 ± 0.5	-0.4 ± 0.6	-0.4 ± 0.7	0.294
	NAB	0.3 ± 0.5	0.0 ± 0.6	-0.3 ± 0.5	0.2 ± 0.9	0.524
Dryness of the vigina	*Control*	1.2 ± 1.2	0.1 ± 0.5	0.0 ± 0.6	0.0 ± 0.6	0.997
	AB	0.8 ± 1.1	0.1 ± 0.5	0.0 ± 0.6	-0.1 ± 0.6	0.909
	NAB	1.3 ± 1.1	0.1 ± 0.7	-0.3 ± 1.1	-0.8 ± 1.0	0.504
**Total MRS score**	*Control*	10.1 ± 5.8	0.2 ± 2.9	-0.5 ± 4.3	-1.1 ± 4.2	0.915
	AB	11.3 ± 5.6a	-2.8 ± 2.8a	-4.0 ± 3.9a	-5.2 ± 4.4b	**0.014**
	NAB	9.9 ± 5.5	-0.9 ± 3.9	-3.6 ± 4.2	-4.2 ± 3.0	0.398

AB: alcoholic beer; NAB: non-alcoholic beer. Results are presented as mean ± SD and mean changes ± SD compared to baseline visit?. Kruskal Wallis followed by post-hoc Dunn's test was used for statistical intragroup comparisons throughtout the intervention. p-value < 0.05.

Table S3. Intragroup analysis of female sex hormone levels before and after intervention.

		Baseline Mean ± SD	6 months Changes ± SD	p-value
LH *(15.9-54.0 U/L)*[1]	*Control*	29.3 ± 10.4	2.2 ± 4.0	0.129
	AB	41.6 ± 15.3	-2.2 ± 6.5	0.175
	NAB	36.9 ± 12.6	1.6 ± 10.3	0.688
FSH *(23-116 U/L)*[1]	*Control*	62.9 ± 21.3	2.3 ± 14.4	0.151
	AB	96.8 ± 42.5	-6.5 ± 10.9	0.039
	NAB	59.9 ± 20.2	3.5 ± 9.6	0.438
E2 *(>37 pg/mL)*[1]	*Control*	51.9 ± 13.0	4.1 ± 23.7	0.685
	AB	35.8 ± 6.6	3.1 ± 20.3	0.815
	NAB	53.0 ± 35.5	-3.0 ± 10.3	0.438
Progesterone *(ng/mL)*	*Control*	0.39 ± 0.27	-0.02 ± 0.29	0.549*
	AB	0.32 ± 0.13	0.02 ± 0.08	0.515
	NAB	0.29 ± 0.05	-0.01 ± 0.09	1.000
T-Total *(10-50 ng/dL)*	*Control*	18.7 ± 10.9	1.6 ± 8.2	0.519
	AB	14.4 ± 8.4	0.4 ± 4.6	0.901
	NAB	15.3 ± 9.4	-2.8 ± 4.8	0.219*
SHBG *(25.0-96.0 nmol/L)*	*Control*	53.5 ± 24.0	0.8 ± 12.6	0.470
	AB	59.1 ± 24.3	-7.4 ± 16.5	0.386
	NAB	63.0 ± 32.6	-8.6 ± 17.8	0.219
TFI *(0.43-8.10)*	*Control*	1.76 ± 2.32	0.13 ± 0.46	0.470
	AB	1.01 ± 0.80	0.07 ± 0.56	0.561
	NAB	1.06 ± 0.79	-0.08 ± 0.30	0.688*
FEI *(nmol/L)*	*Control*	0.46 ± 0.32	0.03 ± 0.21	0.850
	AB	0.27 ± 0.14	0.04 ± 0.11	0.231
	NAB	0.55 ± 0.78	-0.01 ± 0.10	1.000*

[1]Postmenopausal reference values. AB: alcoholic beer; FEI: free estradiol index; FSH: Follicle-stimulating hormone; LH: Luteinizing hormone; NAB: non-alcoholic beer; SHBG: sex hormone-binding globulin; TFI: Free testosterone index; T-Total: Total testosterone. Wilcoxon matched-pair signed-rank test was was used for statistical intragroup comparisons throughtout the intervention. Sing test of matched-pairs was used in asymmetric distributed variables (*).
